# Impact of Enhanced Family Education on BMI Changes in Children and Adolescents With Overweight or Obesity: Study Protocol for a City-Wide Cluster Randomized Controlled Trial

**DOI:** 10.2196/86508

**Published:** 2026-03-26

**Authors:** Rui Hang Zhang, Ting Yu Lu, Si Han Hou, Ya Bin Qu, Qiu Xia Chen, Meng Li, Jiao Wang, Lin Xu

**Affiliations:** 1 School of Public Health, Sun Yat-sen University, Rm 103, School of Public Health Building, No. 74 Zhongshan 2nd Road, Yuexiu District, Guangzhou, 510080, China, 86 20-87335523, 86 20-87330446; 2 Greater Bay Area Public Health Research Collaboration, Guangzhou, China; 3 Guangdong Provincial Center for Disease Control and Prevention, Guangzhou, China; 4 Department of Applied Health Sciences, University of Birmingham, Birmingham, United Kingdom; 5 School of Public Health, University of Hong Kong, Hong Kong, China (Hong Kong)

**Keywords:** enhanced family education, obesity, school, China, randomized controlled trial

## Abstract

**Background:**

The prevalence of obesity among children and adolescents has become a critical global public health issue, particularly in China, with significant increases observed over the past few decades. Despite regular surveillance for overweight and obesity in the past decades, there have been no annual reports provided to parents, resulting in a significant underrecognition of this issue. Early intervention and enhanced family-based educational intervention are necessary to address this growing problem.

**Objective:**

This protocol aims to evaluate the effectiveness of an enhanced family-based educational intervention in reducing overweight and obesity among school-age children and adolescents.

**Methods:**

This study uses a stratified cluster randomized controlled trial design involving 40 schools from 4 cities in Guangdong Province, selected based on economic levels and overweight and obesity prevalence. Schools will be randomly assigned to intervention or control groups. The intervention group receives an enhanced family-based educational intervention on obesity prevention, including health reports and educational materials. The control group continues with regular practices, including routine physical examinations, general health education activities, and school-based physical activity programs. The intervention spans 9 months, followed by a 3-month follow-up. Data on BMI, waist circumference, and waist-to-height ratio are collected at baseline and after 12 months to assess the potential effectiveness of the intervention.

**Results:**

This trial was funded in January 2024 and registered in the Chinese Clinical Trial Registry on November 5, 2024. Recruitment was completed in December 2024, with 20 schools enrolled in each of the intervention and control groups. Baseline data collection was completed during the 2024 fall semester, and follow-up data collection continued through December 2025. Data analysis will start after completion of the 12-month assessments. The study findings are expected to be published in 2026.

**Conclusions:**

This study protocol addresses the urgent need for effective interventions to combat the increasing prevalence of childhood and adolescent obesity in Guangdong Province. Given the significant underrecognition of the need for consistent reporting to parents, the proposed enhanced family-based educational intervention aims to fill this gap by raising parental awareness and promoting healthier lifestyles among children and adolescents. If successful, this approach could significantly reduce the risk of overweight and obesity in Chinese populations, which account for approximately one-fifth of the world’s population. The findings will provide insights into the efficacy of family-centered interventions and underscore the importance of integrating routine parental reporting into existing surveillance programs.

## Introduction

The global prevalence of obesity among children and adolescents has increased nearly 10-fold over the past 4 decades, from 0.7% in 1975 to 5.6% in 2016 among girls and from 0.9% to 7.8% among boys in the same period [1,2]. This prevalence continues to rise, with more than 390 million children and adolescents aged 5 to 19 years being overweight, including 160 million who are living with obesity as of 2022 [3]. While the prevalence of obesity has plateaued at high levels in high-income countries, it is still accelerating in low- and middle-income countries, including China [2,4]. The mean prevalence of overweight and obesity among Chinese children and adolescents increased significantly from 5.3% in 1995 to 20.5% in 2014 [5], and more recent data reported by the latest round of China Chronic Disease and Nutrition Surveillance (2015-2019) indicated that the prevalence among children and adolescents aged 6 to 17 years was approximately 19% for overweight and obesity as of 2019 [6]. Childhood and adolescent obesity can affect both physical and psychological health [3], academic attainment, and quality of life [7,8]. In the longer term, it can also increase the risk of cardiometabolic diseases [9], musculoskeletal problems [10], and cancers in adulthood [11].

Despite the alarming rise in childhood and adolescent obesity, many Chinese parents and caregivers hold the belief that a chubby child is a sign of good health and nutrition, reflecting social and cultural factors where a fuller body is seen as a sign of prosperity and well-being [12]. This perception is compounded by the common belief that children who are overweight will naturally shed the excess weight as they grow older [13]. However, without proper intervention, children who are overweight are more likely to become adults who are overweight or obese, carrying the associated health risks into later life [14,15]. The issue is further complicated by the misconception that obesity primarily affects adults, leading to insufficient attention to the dietary and physical activity needs for children and adolescents [16,17]. Many parents are not fully aware of the serious health risks linked to childhood and adolescent obesity, such as diabetes, hypertension, and psychological issues [18-20]. This lack of awareness contributes to a lack of urgency in addressing and managing weight issues among young people [19,20].

Existing literature underscores the need for early intervention to curb the trajectory of obesity from childhood into adulthood [15,20,21]. Studies have shown that interventions targeting children and adolescents can effectively reduce BMI and improve motor skills [22-24].

Despite regular surveillance and monitoring of overweight and obesity in China, there has been a significant underrecognition of these issues due to the absence of consistent reporting to parents. Furthermore, evidence suggests that interventions with significant family involvement achieve better outcomes in reducing childhood and adolescent obesity [25]. However, the effectiveness of enhanced family-based educational interventions aimed at children and adolescents with overweight or obesity has not yet been fully evaluated. Notably, most studies evaluating obesity interventions in China have not adequately measured the degree of family involvement during implementation [26-32]. Furthermore, these studies were confined to single, high-income cities, such as Shanghai [31], Nanjing [28,32], Guangzhou [30], and Beijing [29], raising concerns about their generalizability to broader, more diverse populations across the country. Therefore, these gaps highlight the need for enhanced family-based educational interventions to raise awareness and promote healthier lifestyles among children and adolescents, together with their families.

This study aims to evaluate the effectiveness of enhanced family-based educational interventions in reducing the prevalence of overweight and obesity among school-aged children and adolescents in Guangdong Province. By integrating routine parental reporting into existing surveillance programs and providing comprehensive family-centered interventions, this study seeks to address the critical gaps in awareness and management of childhood and adolescent overweight and obesity. The findings will contribute valuable insights into effective strategies for obesity prevention and control, ultimately improving the health outcomes for children and adolescents in China.

## Methods

### Study Design

**Figure 1.**
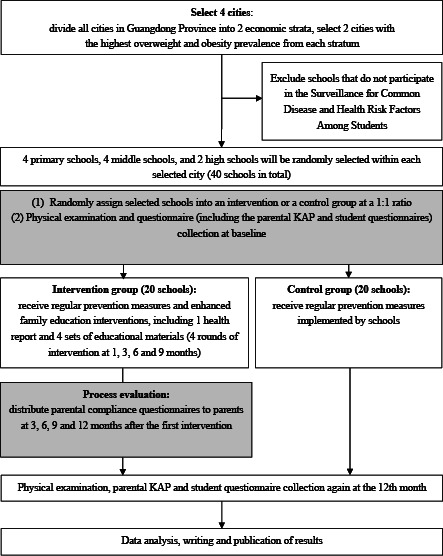
Overview of the study design. KAP: knowledge, attitude, and practice.

This study will use a multistage cluster randomized controlled trial (RCT) design. All cities in Guangdong Province will be divided into 2 economic strata. The 2 cities with the highest prevalence of overweight or obesity will be selected from each stratum. Schools within these cities will be randomly assigned to either the intervention group or the control group. The intervention group will receive enhanced family-based educational interventions, while the control group will continue with regular practices. The study will involve baseline data collection, 4 rounds of interventions, and follow-up assessments over 12 months. Figure 1 shows the flowchart of the study design.

### Setting

The study will be conducted in Guangdong Province, which includes 21 prefecture-level cities. Within these cities, a total of 40 schools (including primary, middle, and high schools) will be selected based on their participation in an initiative called Surveillance for Common Disease and Health Risk Factors Among Students in Guangdong Province. The selected schools will represent a diverse socioeconomic and demographic student population to ensure the generalizability of the study findings. The intervention will be implemented in both urban and rural settings to capture the varying dynamics of childhood and adolescent obesity across different environments.

### Stratification, Randomization, and Allocation Concealment

Stratified randomization will be used, considering the comparability between the intervention and control groups. First, all cities in Guangdong Province will be divided into 2 economic strata. The 2 cities with the highest prevalence of overweight or obesity will be selected from each stratum. Second, schools will be stratified according to school types, with 4 primary schools, 4 middle schools, and 2 high schools being selected in each city. Subsequently, the selected schools within each stratum will be randomly assigned in a 1:1 ratio to either the intervention or control group. To ensure rigorous allocation concealment, a random allocation sequence will be generated by an independent statistician blinded to the participating schools. Opaque, sealed envelopes containing group assignments will be prepared by staff not involved in intervention implementation, and allocations will be disclosed only after completion of school enrollment.

### Inclusion and Exclusion Criteria

Participants in this study will be school-aged children and adolescents from Guangdong Province who meet the following criteria: aged between 8 and 17 years and enrolled in grades 4 and 5 (primary school), grades 7 and 8 (middle school), and grades 10 and 11 (high school). They must be identified as overweight or obese based on the Screening for Overweight and Obesity Among School-Age Children and Adolescents (WS/T 586‐2018) standards during the baseline assessment. Additionally, both students and their parents or guardians must provide informed consent to participate in the study.

Participants will be excluded if they meet any of the following criteria: refusal by students or their parents or guardians to participate in the study; presence of mental health conditions, intellectual disabilities, or other factors that may impede understanding or cooperation with the study procedures; and presence of severe illnesses or undergoing major medical treatments, such as dialysis or chemotherapy, which may affect the study outcomes.

### Sample Size

Assuming an effect size (Cohen *d*) of 0.1, an SD of 0.05, a significance level (α) of .05, and a power (1-*β*) of .9, with an intracluster correlation coefficient of 0.01, an average cluster size (*m*) of 160 students, and an overweight and obesity prevalence rate of 20%, the required sample size *n* for each cluster (school) was calculated using the following Cohen *d* formula:


(1) $$n={\left(\frac{z_{1-\frac{a}{2}}+z_{1-\beta }}{d}\right)}^{2}\times 2\times {\left(SD\right)}^{2}$$

Considering that the sample size for a cluster RCT needs to be adjusted based on the intracluster correlation coefficient, the adjusted sample size n was calculated. By multiplying the adjusted sample size *n* by the number of clusters (schools), the total sample size was estimated to be 539 students. Accounting for a 20% dropout rate, the total sample size required is approximately 674 students.

### Intervention and Control

The intervention group will receive an enhanced family-based educational intervention in addition to the regular prevention measures implemented by schools. Each student and their parents will receive 1 health report (Multimedia Appendix 1) and 4 sets of educational materials, which will be developed by the Guangdong Provincial Center for Disease Control and Prevention and delivered by schools. Each set includes an enhanced health education booklet, a family weight management action guideline, and an advocacy letter (Multimedia Appendix 2). Details of these intervention components are shown in Table 1. Participants in the control group will only receive the regular prevention measures implemented by their schools. These measures may include routine physical examinations, general health education activities, and school-based physical activity programs, but no additional educational materials or personalized health reports will be provided to the control group.

**Table 1.** Description of the enhanced family-based educational intervention components.

Intervention component	When it was delivered	What was delivered
Health report	Within 1 month of the baseline survey (ie, the first round of the intervention)	Information on the student's height, weight, waist circumference, BMI, and their overweight or obesity status
Advocacy letter	Within 1 month of the baseline survey (ie, the first round of the intervention), and at 3, 6, and 9 months after the first intervention	Information on the impact of being overweight and obese on the physical and brain development of students and the “Three 1/2s” initiative: increasing the daily intake of fresh fruits and vegetables by half, reducing the daily intake of sugary drinks and fried foods by half, and increasing daily outdoor physical activity duration by half
Enhanced health education booklet	Within 1 month of the baseline survey (ie, the first round of the intervention), and at 3, 6, and 9 months after the first intervention	Information on the short-term and long-term health risks associated with childhood and adolescent overweight and obesity, healthy dietary habits to prevent obesity, physical activity recommendations, BMI calculation methods, and screening standards for childhood and adolescent overweight and obesity
Family weight management action guideline	Within 1 month of the baseline survey (ie, the first round of the intervention), and at 3, 6, and 9 months after the first intervention	Details of the implementation of the “Three 1/2s” initiative, methods for monitoring children and adolescents' growth, and monthly checklists for tracking adherence to the intervention

### Study Outcomes

Primary outcomes are the changes in BMI and standardized BMI (zBMI) over a 12-month period. These outcomes will provide a direct measure of the intervention’s effectiveness in reducing overweight and obesity among the participants. The change in BMI will be assessed from baseline to the end of the study, while the change in zBMI will account for age and sex differences, offering a more nuanced understanding of the weight status changes in the school-aged children and adolescents.

Secondary outcomes include the incidence and recovery rates of both general and central obesity; changes in standardized waist-to-height ratio (zWHtR) and standardized waist circumference (zWC); and changes in parental knowledge, attitude, and practice (KAP) scores (Multimedia Appendix 3) [33]. Specifically, the incidence of general obesity will be determined by newly diagnosed students with obesity at the end of the study among participants who are not obese at baseline, and the recovery rate will be calculated based on the number of students who transition from obesity to nonobesity status. Similarly, the incidence and recovery rates of central obesity will be assessed. Additional secondary outcomes involve changes in zWHtR and zWC over 12 months. The staff who measure the children’s weight and height will be blinded to the school allocation. Furthermore, KAP scores will be assessed at the start of the intervention and at 3, 6, 9, and 12 months after the intervention to gauge improvements in health literacy regarding childhood and adolescent overweight and obesity. These comprehensive outcomes will help elucidate the effectiveness of the enhanced family-based educational interventions and their impact on health behaviors and literacy.

Process evaluation will be used to evaluate the feasibility and acceptability of the trial by assessing parental compliance. Specifically, parent questionnaires (Multimedia Appendix 4) are administered at 3, 6, 9, and 12 months after the intervention to evaluate adherence to dietary and physical activity recommendations.

### Data Collection

Data collection will include both questionnaire surveys and anthropometric measurements, carried out at the beginning and end of the study. The 2024 Student Health Status and Influencing Factors Survey, integrated with the provincial Surveillance for Common Disease and Health Risk Factors Among Students project, will be administered before the study begins and after it concludes. Both students and their parents will complete the survey, which is provided by national authorities and includes versions for primary and secondary school students. The survey covers general demographic characteristics, dietary habits, physical activity, smoking and drinking behaviors, use of eyes outside of school, electronic screen use, outdoor activities, and sleep habits. Additionally, parental KAP questionnaires for the intervention group will be collected at baseline and 3, 6, 9, and 12 months, while those for the control group will be obtained at baseline and after 12 months. Nutritional status monitoring will include measurements of height, weight, and waist circumference following standardized procedures with light clothing and without shoes. BMI will be calculated as weight in kilograms divided by height in meters squared (kg/m^2^). Waist circumference will be measured horizontally at the midpoint between the last rib and the top of the iliac crest. General obesity will be determined by sex- and age-specific BMI cutoff values according to the Screening Standard for Overweight and Obesity Among School-Age Children and Adolescents (WS/T 586-2018). Central obesity will be based on the 90th percentile of waist circumference by sex and age according to the High Waist Circumference Screening Threshold for Children and Adolescents Aged 7-18 Years (WS/T 611-2018). Data collected from paper-based questionnaires will be independently double-entered by 2 trained university students and subsequently entered into an electronic database.

### Statistical Analysis

The collected data will be analyzed to evaluate the effectiveness of the enhanced family-based educational interventions. Descriptive statistics will summarize baseline characteristics of the intervention and control groups, with means (SDs) or medians (IQRs) for continuous variables and frequencies (percentages) for categorical variables. Comparisons between groups will be made using independent sample 2-tailed *t* tests or Wilcoxon rank-sum tests for continuous variables and chi-square tests or Fisher exact tests for categorical variables. Multivariable regression analyses will adjust for potential confounders and assess the intervention’s independent effects on primary and secondary outcomes, including changes in BMI, zBMI, zWHtR, and zWC over 12 months. Parental KAP scores will be the sum of scores in each questionnaire entry, which will be scored on a scale of 1 to 4 depending on the option. KAP scores from the intervention group will be assessed every 3 months, and the difference between KAP scores at the start and the end of the study will be used to evaluate changes in health literacy.

To address the handling of missing data in our study, we will use a combination of intention-to-treat, per-protocol, and complete case analyses. The intention-to-treat analysis will include all participants as randomized to account for nonadherence and dropouts, thereby preserving the randomization’s integrity. The per-protocol analysis will focus on participants who complete the intervention according to the study protocol, offering insights into the efficacy under ideal adherence. Additionally, we will conduct a complete case analysis, which considers only those participants with complete data on all variables of interest. This approach will allow us to maintain the rigor of our statistical evaluation while acknowledging the limitations associated with missing data.

### Ethical Considerations

This study protocol was reviewed and approved by the institutional review board of the School of Public Health, Sun Yat-sen University (approval number 2024-160). Informed consent will be obtained from all participants and their parents or guardians prior to enrollment in the study. All procedures will be conducted in accordance with the ethical standards of the responsible committee and the World Medical Association Declaration of Helsinki. Participant confidentiality will be ensured by assigning unique study IDs, and privacy will be strictly protected. Any participant who experiences harm related to trial participation will receive appropriate medical care and compensation.

## Results

This trial was funded in January 2024. The study recruitment is scheduled to be completed before December 2024. The intervention will be integrated with the 2024 Surveillance for Common Disease and Health Risk Factors Among Students in Guangdong Province initiative. Baseline data will be collected during the 2024 and 2025 fall semester. Within 1 month, the local Center for Disease Control and Prevention will provide the intervention group with health reports and educational materials (including an enhanced health education booklet, a family weight management action guideline, and an advocacy letter). Educational materials will also be sent to the intervention group at 3, 6, and 9 months after the first intervention. Health reports are provided at the first intervention. The control group will continue with regular school-based prevention measures. The total intervention duration lasts 9 months, with 4 rounds of intervention. Table 2 shows the timeline of the study implementation. Data collection is scheduled to conclude in December 2025. Data analysis will begin after completion of the 12-month assessments. At the time of manuscript submission, baseline data collection was complete, with 20 schools enrolled in each of the intervention and control groups. Descriptive and analytic results are expected to be published in 2026.

**Table 2.** Timeline of the study implementation.

Task	November-December 2024		January-December 2025											
	November	December	January	February	March	April	May	June	July	August	September	October	November	December
Ethics approval and clinical trial registration	✓^a^													
Sampled school and class determination	✓^a^													
Staff training	✓^a^													
Student survey questionnaire distribution		✓^b^												✓^b^
Parental KAP^c^ questionnaire distribution		✓^b^			✓^d^			✓^d^			✓^d^			✓^b^
Student health check-up		✓^b^												✓^b^
Health report distribution		✓^d^												
Enhanced health education booklet distribution		✓^d^			✓^d^			✓^d^			✓^d^			
Advocacy letter distribution		✓^d^			✓^d^			✓^d^			✓^d^			
Family weight management action guideline distribution		✓^d^			✓^d^			✓^d^			✓^d^			
Parental compliance questionnaire distribution					✓^d^			✓^d^			✓^d^			✓^d^

a Preliminary preparation work.

b Tasks for all participants.

c KAP: knowledge, attitude, and practice.

d Tasks only for the intervention group.

## Discussion

### Hypothesized Findings

The enhanced family-based educational intervention described here is anticipated to be effective for obesity control, reducing the prevalence of overweight and obesity among children and adolescents in Guangdong Province. We hypothesize that, compared with the control group, the intervention group will show significantly more favorable changes in BMI, zBMI, zWHtR, and zWC over the 12-month period. We also anticipate a significantly lower incidence of general obesity and a higher recovery rate in the intervention group. Furthermore, the intervention is expected to improve parental health literacy. This study integrates surveillance feedback with an enhanced family-based educational intervention, aiming to generate critical evidence to inform obesity prevention and control policies in China.

### Comparison to Prior Work

Previous intervention studies with family involvement have been predominantly conducted in single, high-income cities [28-32], limiting the generalizability of their findings to populations with different economic levels in China. Furthermore, these studies overlooked standardized family guidelines and the timely feedback of surveillance results to parents [34], neglecting elements essential for sustained engagement. To address these gaps, this study uses a multicity cluster RCT design across cities of varying economic levels. Additionally, we will provide structured educational materials, including the actionable “Three 1/2s” initiatives and child health reports for parents, aiming to facilitate practical implementation and enhance family engagement.

### Strengths and Limitations

This study has some strengths, including randomly selecting 4 cities from 2 economic strata to enhance representativeness. Additionally, schools will serve as the unit of intervention, rather than individuals, to minimize contamination between groups and improve implementation feasibility. Several limitations should be acknowledged. First, although a relatively short follow-up period may limit the detection of significant intervention effects on the control of overweight or obesity, we implemented 4 rounds of enhanced intervention, with continuous process evaluations and monitoring of parental health literacy. Second, although the study protocol does not include tailored, culturally adaptive materials, analyzing the data for differential effects across socioeconomic subgroups may provide insights to inform future refinements of intervention strategies. Finally, the anthropometric measurements will be performed only at baseline and at the end of the study based on the provincial Surveillance for Common Disease and Health Risk Factors Among Students project. The 2-point measurements restrict our ability to capture the dynamic trajectories of nutritional status during the study. Future studies incorporating more detailed and frequent measurements are warranted to better characterize the temporal trajectories of the intervention effects.

### Future Directions

This study aims to assess the effectiveness of a cost-effective family-based educational intervention to address childhood obesity in China. If proven effective, this framework could provide a scalable solution for nationwide implementation. Future research may explore tailored strategies across diverse socioeconomic and urban-rural contexts to enhance intervention effectiveness. In addition, identifying the optimal frequency of parental reporting would be valuable for maximizing long-term adherence.

### Dissemination Plan

Study results will be published in peer-reviewed journals and presented at conferences and relevant stakeholder engagement activities. No information that can identify participants will be included in any publication. Participants who explicitly express a wish to be informed about the study outcome will be contacted and provided with an article or poster containing a lay summary of findings.

### Conclusions

This cluster RCT will evaluate the effectiveness of an enhanced family-based educational intervention in children and adolescents with overweight or obesity. By integrating parental reporting into the existing provincial health surveillance system, the study aims to bridge the critical gap between school-based screening and family-based management, addressing widespread underrecognition of childhood obesity. The findings will provide evidence on the efficacy and feasibility of this scalable, family-based model. If proven effective, this approach could be incorporated into national surveillance programs, offering a sustainable public health strategy to curb the growing obesity epidemic.

## Supplementary material

10.2196/86508 Multimedia Appendix 1 Overweight and obesity screening report.

10.2196/86508 Multimedia Appendix 2 “Wise drinking and smart movement” advocacy.

10.2196/86508 Multimedia Appendix 3 Parental knowledge, attitude, and practice survey.

10.2196/86508 Multimedia Appendix 4 Parental compliance evaluation questionnaire.

10.2196/86508 Checklist 1 SPIRIT 2025 checklist.

## Abbreviations

KAP: knowledge, attitude, and practice
RCT: randomized controlled trial
zBMI: standardized BMI
zWC: standardized waist circumference
zWHtR: standardized waist-to-height ratio
