# Supplementary material for: Impact of Enhanced Family Education on BMI Changes in Children and Adolescents With Overweight or Obesity: Study Protocol for a City-Wide Cluster Randomized Controlled Trial
Source: JMIR Res Protoc. 2026 Mar 26;15:e86508. doi: 10.2196/86508 (PMC13021105; doi:10.2196/86508)
Supplement: Multimedia Appendix 1 [file resprot-v15-e86508-s001.docx]

**Appendix 1**

**Overweight and Obesity Screening Report**

School: Class:

Dear Parents,

Your child has participated in the "Wise Drinking and Smart Movement" program for overweight and obesity intervention. Your child’s anthropometric indicators, current status, and related recommendations are provided for your reference as below:

1. **Anthropometric indicators**

Height: *** centimeters (cm)

Weight: *** kilograms (kg)

Waist circumference: *** centimeters (cm)

BMI: *** kg/m^2^

1. **Current status**

Preliminary assessment of your child:

(1) Normal BMI (2) Normal waist circumference

(3) General overweight (4) General obesity

(5) Central overweight (6) Central obesity

1. **Related Recommendations**

(1) Help your child develop a healthy lifestyle by maintaining a balanced diet with plenty of vegetables, fruits, whole grains, and high-quality proteins, while reducing intake of sugar-sweetened beverages and fried foods.

(2) Ensure at least 2 hours of outdoor activity per day, limit screen time to no more than 1 hour per day, and encourage your child to participate in household chores.

(3) Regularly monitor your child’s height and weight changes on a weekly basis.

Thanks for your attention and support!

Guangdong Provincial Center for Disease Control and Prevention

1 December 2024
