# Supplementary material for: Impact of Enhanced Family Education on BMI Changes in Children and Adolescents With Overweight or Obesity: Study Protocol for a City-Wide Cluster Randomized Controlled Trial
Source: JMIR Res Protoc. 2026 Mar 26;15:e86508. doi: 10.2196/86508 (PMC13021105; doi:10.2196/86508)
Supplement: Multimedia Appendix 2 [file resprot-v15-e86508-s002.docx]

**Appendix 2**

**"Wise Drinking and Smart Movement" Advocacy**

Dear Parents,

The government attaches great importance to the health problems of children and adolescents. Departments of education or disease control and prevention pay much attention to the health status of students and we hope to work with you to ensure that they can possess a healthy and happy childhood. We have recently conducted a physical examination for your child and **the results indicate that your child is currently overweight or obese**. It not only has impact on body shape, but may also have a series of effects on the current and future health of children.

Medical evidence shows that obesity may lead to a variety of health problems such as type 2 diabetes, hypertension, hyperlipidemia. In addition, obesity can have negative impacts on child’s brain function development, affecting their attention, memory and study performance. Therefore, it is important to prevent and control childhood and adolescence obesity as early as possible.

In order to improve your child’s health, we advocate for **"Wise Drinking and Smart Movement"** initiative which aims to help children change overweight or obese status and grow healthily under scientific diet and exercise guidance. We advocate for **"Three 1/2s"** initiatives including:

1. **Increase the daily intake of fresh fruits and vegetables by half.** Fresh fruits and vegetables are rich in vitamins, minerals and dietary fiber which can not only be helpful to control weight, but also enhance immunity and promote physical and brain development. We recommend you increase the proportion of these healthy foods in your child's daily diet.

2. **Reduce the daily intake of sugary drinks and fried foods by half.** Sugary drinks and fried foods are usually high in calories and low in nutritional value, which is one of the main factors leading to obesity. We recommend you decrease your child's intake of these unhealthy foods and choose healthier alternatives such as water and low-fat milk.

3. **Increase daily outdoor physical activity duration by half.** Regular outdoor activity can not only be helpful to control weight, but also improve cardiorespiratory function, metabolic and mental health. Therefore, we recommend you urge your child to increase their daily outdoor physical activity duration by at least half.

The health education booklet includes the short-term and long-term health risks associated with childhood and adolescence overweight and obesity, healthy dietary habits to prevent obesity, physical activity recommendations, body mass index (BMI) calculation methods, and screening standards for childhood and adolescence overweight and obesity. We hope you will read it with your child and help them develop healthy dietary and activity habits. If you have any questions or need more information, please feel free to contact us. Let us work together to pay attention to children's healthy future.

Wish you and your family good health and happy life!

Disease Control and Prevention Administration of Guangdong Province

Department of Education of Guangdong Province

Guangdong Provincial Center for Disease Control and Prevention

1 December 2024
