# Supplementary material for: Impact of Enhanced Family Education on BMI Changes in Children and Adolescents With Overweight or Obesity: Study Protocol for a City-Wide Cluster Randomized Controlled Trial
Source: JMIR Res Protoc. 2026 Mar 26;15:e86508. doi: 10.2196/86508 (PMC13021105; doi:10.2196/86508)
Supplement: Multimedia Appendix 3 [file resprot-v15-e86508-s003.docx]

**Appendix 3**

**Parental Knowledge, Attitude, and Practice Survey**

Dear Parents,

The government attaches great importance to the health problems of children and adolescents. Departments of education or disease control and prevention pay much attention to the health status of students and we hope to work with you to ensure that they can possess a healthy and happy childhood. To better understand your knowledge about overweight and obesity in children and adolescents, please fill out the following questions based on your actual situation. Thank you very much for your support and cooperation!

Disease Control and Prevention Administration of Guangdong Province

Department of Education of Guangdong Province

Guangdong Provincial Center for Disease Control and Prevention

**Basic Information**

1. ID Number: □□ □□ □□ □ □□ □□ □□□□
2. Bar Code: _____

**Knowledge: Do you think the following statements are correct?**

1. Obesity in children and adolescents increases the risk of diabetes.
   1. Completely correct
   2. Mostly correct
   3. Not very correct
   4. Completely wrong
2. Obesity in children and adolescents increases the risk of bone and joint damage.
   1. Completely correct
   2. Mostly correct
   3. Not very correct
   4. Completely wrong
3. Obesity in children and adolescents increases the risk of fatty liver.
   1. Completely correct
   2. Mostly correct
   3. Not very correct
   4. Completely wrong
4. Obesity in children and adolescents can cause high blood pressure.
   1. Completely correct
   2. Mostly correct
   3. Not very correct
   4. Completely wrong
5. Obesity in children and adolescents can lead to psychological problems.
   1. Completely correct
   2. Mostly correct
   3. Not very correct
   4. Completely wrong
6. Obesity in children and adolescents affects relationships with classmates.
   1. Completely correct
   2. Mostly correct
   3. Not very correct
   4. Completely wrong
7. Obesity in children and adolescents is related to abnormal blood lipids.
   1. Completely correct
   2. Mostly correct
   3. Not very correct
   4. Completely wrong

**Attitude/Belief: Do you agree with the following statements?**

1. Eating fast food can easily lead to obesity.
   1. Completely agree
   2. Mostly agree
   3. Do not agree much
   4. Completely disagree
2. Drinking too much juice can lead to weight gain.
   1. Completely agree
   2. Mostly agree
   3. Do not agree much
   4. Completely disagree
3. Drinking too many probiotic drinks (like Yakult) can lead to weight gain.
   1. Completely agree
   2. Mostly agree
   3. Do not agree much
   4. Completely disagree
4. A single type of food is not good for health.
   1. Completely agree
   2. Mostly agree
   3. Do not agree much
   4. Completely disagree
5. Drinking dairy products daily is good for health.
   1. Completely agree
   2. Mostly agree
   3. Do not agree much
   4. Completely disagree
6. Eating night snacks easily leads to weight gain.
   1. Completely agree
   2. Mostly agree
   3. Do not agree much
   4. Completely disagree
7. Controlling weight during pregnancy can prevent obesity in children and adolescents.
   1. Completely agree
   2. Mostly agree
   3. Do not agree much
   4. Completely disagree
8. Adding complementary foods too early in infancy increases the risk of obesity.
   1. Completely agree
   2. Mostly agree
   3. Do not agree much
   4. Completely disagree
9. Breastfeeding can prevent obesity in children and adolescents.
   1. Completely agree
   2. Mostly agree
   3. Do not agree much
   4. Completely disagree
10. Childhood and adolescence obesity is not a disease.
    1. Completely agree
    2. Mostly agree
    3. Do not agree much
    4. Completely disagree
11. Only adults need to pay attention to their weight.
    1. Completely agree
    2. Mostly agree
    3. Do not agree much
    4. Completely disagree
12. It is okay for children to be a bit overweight.
    1. Completely agree
    2. Mostly agree
    3. Do not agree much
    4. Completely disagree

**Practice: Please answer the following questions based on your actual situation.**

1. Are you able to provide a balanced diet for your child?
   1. Every day
   2. Most of the time
   3. Occasionally
   4. Hardly ever
2. Do you read the nutritional content labels (e.g., calories, fat, protein) when buying packaged foods?
   1. Every day
   2. Most of the time
   3. Occasionally
   4. Hardly ever
3. Do you maintain a healthy diet yourself (e.g., low consumption of salt, low consumption of oil)?
   1. Every day
   2. Most of the time
   3. Occasionally
   4. Hardly ever
4. Do you encourage your child to participate in food preparation?
   1. Every day
   2. Most of the time
   3. Occasionally
   4. Hardly ever
5. Do you regularly measure your child's height and weight?
   1. More than once a week
   2. More than once a month
   3. Occasionally
   4. Never
6. Can you ensure your child consumes a certain amount of fruits and vegetables daily?
   1. Every day
   2. Most of the time
   3. Occasionally
   4. Hardly ever
7. Are you aware of how to calculate Body Mass Index (BMI)?
   1. Very aware
   2. Somewhat aware
   3. Not very aware
   4. Not aware at all
8. Can you ensure your child exercises moderately (e.g., walking, jogging) for at least 1 hour daily?
   1. Every day
   2. Most of the time
   3. Occasionally
   4. Hardly ever
9. Do you pay attention to the information in the school's annual health examination reports?
   1. Every time
   2. Most of the time
   3. Occasionally
   4. Never
10. Can you ensure your child's screen time (e.g., watching TV, using mobile phones, iPads) does not exceed 2 hours daily?
    1. Every time
    2. Most of the time
    3. Occasionally
    4. Hardly ever
