# Supplementary material for: Impact of Enhanced Family Education on BMI Changes in Children and Adolescents With Overweight or Obesity: Study Protocol for a City-Wide Cluster Randomized Controlled Trial
Source: JMIR Res Protoc. 2026 Mar 26;15:e86508. doi: 10.2196/86508 (PMC13021105; doi:10.2196/86508)
Supplement: Multimedia Appendix 4 [file resprot-v15-e86508-s004.docx]

**Appendix 4**

**Parental Compliance Evaluation Questionnaire**

Dear Parents,

The government attaches great importance to the health problems of children and adolescents. Departments of education or disease control and prevention pay much attention to the health status of students and we hope to work with you to ensure that they can possess a healthy and happy childhood. Thank you very much for actively participating in the "Wise Drinking and Smart Movement" initiative. To better understand your participation, please fill out the following questions based on your actual situation after receiving the health educational materials. We greatly appreciate your support and cooperation!

Disease Control and Prevention Administration of Guangdong Province

Department of Education of Guangdong Province

Guangdong Provincial Center for Disease Control and Prevention

**Basic Information**

1. ID Number: □□ □□ □□ □ □□ □□ □□□□
2. Bar Code: _____

**Reading of Health Educational Materials**

1. Did you receive your child's health report?
   - Yes
   - No
2. Did you read the health education booklet on preventing overweight and obesity in children and adolescents?
   - Yes
   - No
3. Did you read the "Wise Drinking and Smart Movement" advocacy letter?
   - Yes
   - No

**Parental Attitude**

1. Do you think the health report is helpful in understanding your child's health status?
   - Very helpful
   - Somewhat helpful
   - Not helpful
2. Do you think the "Wise Drinking and Smart Movement" advocacy letter is helpful in improving your child's health?
   - Very helpful
   - Somewhat helpful
   - Not helpful
3. Do you think the health education booklet is helpful in improving your child's health?
   - Very helpful
   - Somewhat helpful
   - Not helpful
4. Are you willing to continue receiving similar health educational materials (including health reports, advocacy letters, and health education booklets)?
   - Yes
   - No

**Implementation**

1. In the past three months, were you able to supervise your child daily to follow the dietary and exercise recommendations in the health education materials?
   - Every day
   - Most of the time
   - Occasionally
   - Hardly ever
2. Since receiving the health education materials, have you increased your child's daily intake of fresh vegetables and fruits?
   - Yes, by 1/2 or more
   - Yes, by less than 1/2
   - No change
   - Decreased
3. Since receiving the health education materials, have you reduced your child's daily intake of sugary drinks and fried foods?
   - Yes, by 1/2 or more
   - Yes, by less than 1/2
   - No change
   - Increased
4. Since receiving the health education materials, have you increased your child's daily outdoor exercise time?
   - Yes, by 1/2 or more
   - Yes, by less than 1/2
   - No change
   - Decreased
5. Do you find it difficult to implement these initiatives?
   - No (Skip question 15)
   - Yes
6. What do you think is the biggest difficulty in implementing these recommendations? (You can choose multiple answers)
   - No time
   - Child's non-cooperation
   - Do not recognize the benefits of these initiatives
   - Do not know how to implement these initiatives
   - Others: _____

**Feedback**

- Do you have any suggestions or opinions about this intervention project?
- What do you think is a good way to adhere to the implementation of the "Wise Drinking and Smart Movement" initiative?
